# Unveiling the Multifaceted Management of Oral Mucositis in Cancer Patients: A Narrative Review

**DOI:** 10.7759/cureus.55213

**Published:** 2024-02-29

**Authors:** Sangavi R, Indumathy Pandiyan

**Affiliations:** 1 Department of Oral Medicine, Radiology, and Special Care Dentistry, Saveetha Dental College and Hospitals, Saveetha Institute of Medical and Technical Science, Saveetha University, Chennai, IND; 2 Department of Public Health Dentistry, Saveetha Dental College and Hospitals, Saveetha Institute of Medical and Technical Sciences, Saveetha University, Chennai, IND

**Keywords:** quality of life, chemotherapy, radiation, reactive oxygen species, inflammation, oral mucositis

## Abstract

Oral mucositis (OM) is a major and common adverse reaction to cancer treatment, occurring in all patients who undergo radiation therapy or chemotherapy that includes the mucosal areas of the oral and oropharyngeal region. The pathophysiology of OM remains incompletely understood, and there are many unanswered questions about the risk factors for developing OM. Multidisciplinary clinicians and researchers must collaborate to better understand and expand treatment strategies for OM and other inflammatory conditions in oncology. This will lead to the development of more effective treatments and reduce the burden of OM in cancer patients. This article comprehensively reviews the risk factors and patient factors associated with OM, its pathogenesis, clinical presentation, grading, and management.

## Introduction and background

Oral mucositis (OM) is a severe and debilitating condition that is a common complication of cancer treatment, including radiotherapy, chemotherapy, targeted therapy, and hematopoietic stem cell transplantation (HSCT) [1]. Apart from the aforementioned causes, some additional factors can increase the risk of developing OM which include compromised oral hygiene, the use of tobacco products, consumption of alcohol, dehydration, nutritional deficiencies, immune suppression, and certain medical conditions, such as diabetes and acquired immunodeficiency syndrome. While not directly causing OM, these factors can impair the healing process and weaken the immune system, making patients more susceptible to developing it. It is characterized by inflammation and ulceration of the oral mucosa, which can lead to pain, difficulty eating and drinking, and an increased risk of infection [2]. The pathophysiology of OM is complex and has not been fully understood. However, it is thought to be caused by a combination of factors, including direct damage to the oral mucosa by radiation or chemotherapy as well as an inflammatory response. OM can have a significant impact on the quality of life of cancer patients and can even delay or interrupt treatment. It is therefore important to diagnose and manage OM early and effectively [3]. An interdisciplinary team of healthcare professionals, including dentists, oncologists, nurses, and dietitians, plays a crucial role in the management of OM. This team can provide patients with comprehensive care, including pain management, nutritional support, and oral hygiene education [4]. By working together, the interdisciplinary team can help patients to cope with the symptoms of OM and reduce the risk of complications. This can lead to improved patient well-being and outcomes.

## Review

Epidemiology

OM is a common and debilitating complication of cancer treatment, such as chemotherapy and radiation therapy, affecting up to 90% of patients with head and neck cancer. The severity of OM in head and neck cancer patients depends on a number of factors, including the type and dose of chemotherapy and radiation therapy, the location of the tumor, and the patient's overall health status [5]. Patients with head and neck cancer who receive both radiation therapy and chemotherapy are at a particularly high risk of developing severe OM. A study found that patients who received chemotherapy or underwent bone marrow transplantation had a 76% risk of developing mucositis. Patients with poor nutritional status and oral hygiene are also more susceptible to developing OM [6].

Pathophysiology 

The pathophysiology of OM caused by chemotherapy and radiotherapy is a complex process that begins with tissue injury. This process was described in a five-phase model by Sonis [5].

Phase 1: Initiation of Tissue Injury

**Figure 1:**
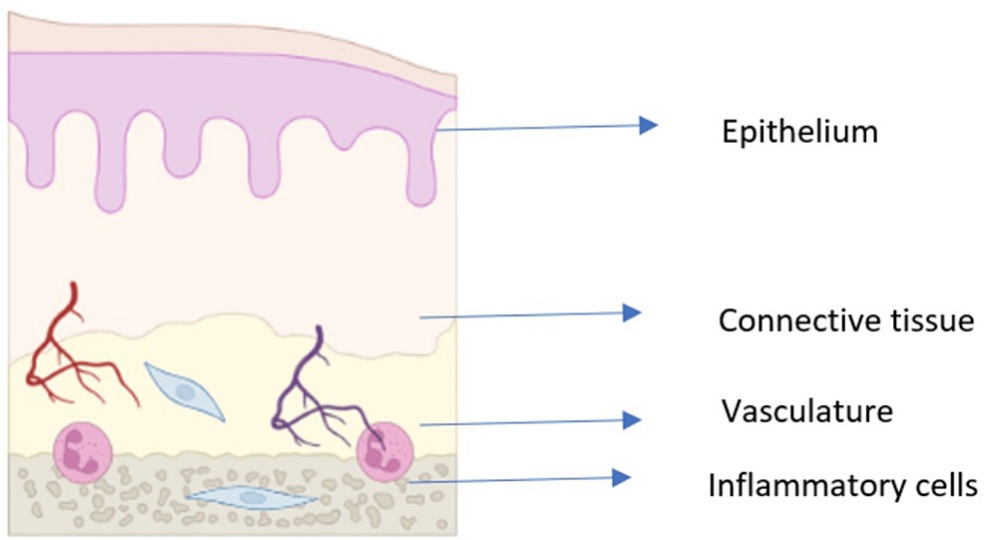
Normal epithelium of the oral cavity Image credits: Dr. Sangavi R

**Figure 2:**
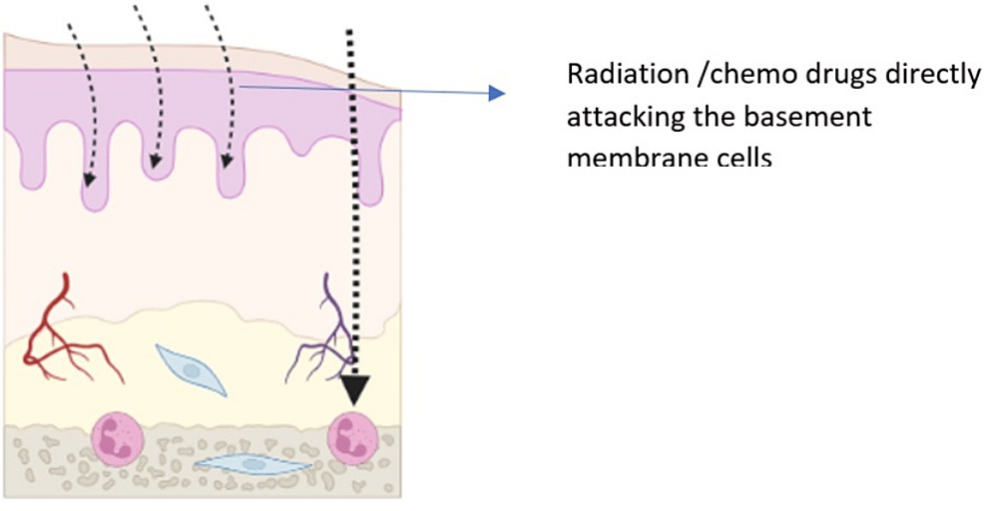
Phase 1-initiation of tissue injury Image credits: Dr. Sangavi R

Radiation and/or chemotherapy can directly damage cells in the basal epithelial layer of the oral mucosa, leading to cell lysis and the generation of reactive oxygen species (ROS), also known as free radicals. ROS are highly reactive molecules that can cause significant cellular damage, and they are believed to play a role in the initiation of mucosal injury. In addition to direct cell damage, radiation and chemotherapy can also induce apoptosis (programmed cell death) in epithelial cells. Apoptosis is a normal cellular process, but it can be accelerated by radiation and chemotherapy, leading to further tissue injury. Another important factor in the initiation of OM is the disruption of the basement membrane, which is a thin layer of extracellular matrix that separates the epithelium from the underlying connective tissue. The basement membrane plays a vital role in supporting the epithelium and protecting it from damage. Radiation and chemotherapy can both damage the basement membrane, making the epithelium more susceptible to injury. Figure 1 represents the normal epithelium of the oral mucosa, and Figure 2 represents the radiation and chemotherapy toxic substance invading the basement membrane and the immune cells in the submucosa.

Phase 2: Messaging and Signaling

In addition to direct cell death and the production of ROS, radiation and chemotherapy can also trigger inflammation through the production of signaling molecules. ROS can activate second messengers, which transmit signals from the receptors on the cell surface to the cell interior. This leads to the overexpression of pro-inflammatory cytokines, which in turn promote cell death. Some of the key pro-inflammatory cytokines involved in OM include tumor necrosis factor-alpha (TNF-α), interleukin-1 beta (IL-1β), and interleukin-6 (IL-6). These cytokines can damage cells directly and can also recruit the immune cells to the site of injury. The immune cells then release additional cytokines and other inflammatory mediators, which further amplify the inflammatory response. The messaging and signaling phase of OM is a critical step in the development of the disease. By understanding the signaling pathways involved, researchers are developing new therapies that could target and disrupt these pathways, preventing inflammation and tissue damage.

Phase 3: Signaling and Amplification

**Figure 3:**
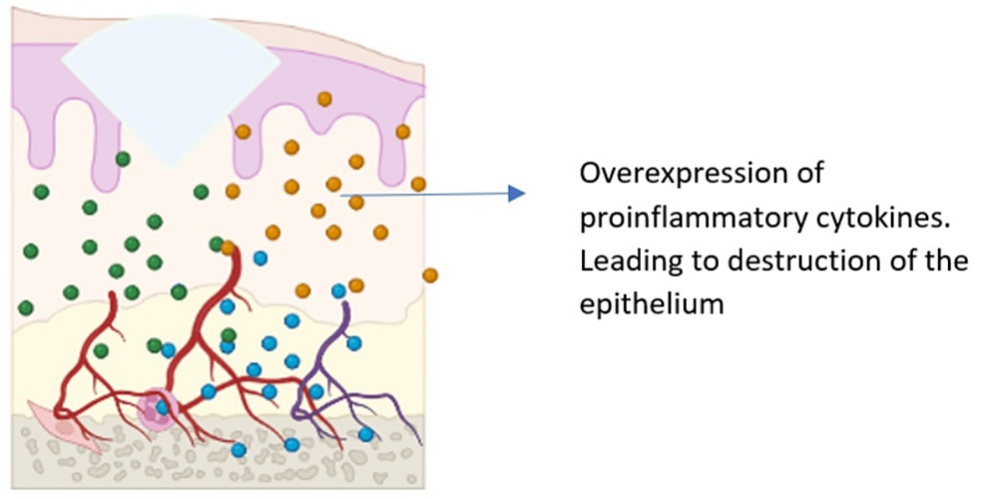
Phases II and III-signaling and amplification leading to cell death and tissue injury Image credits: Dr. Sangavi R

Excessive production of pro-inflammatory cytokines, such as TNF-α, not only damages mucosal cells but also stimulates molecular pathways that amplify mucosal injury. TNF-α is a particularly important pro-inflammatory cytokine in OM. It can damage cells directly and can also activate other signaling pathways that promote inflammation and tissue injury. For example, TNF-α can activate the nuclear factor kappa B (NF-κB) signaling pathway, which leads to the overexpression of additional pro-inflammatory cytokines. The signaling and amplification phase of OM is a vicious cycle that can lead to significant tissue damage. If not controlled, this inflammation can lead to ulceration and other complications (Figure 3).

Phase 4: Ulceration and Inflammation 

**Figure 4:**
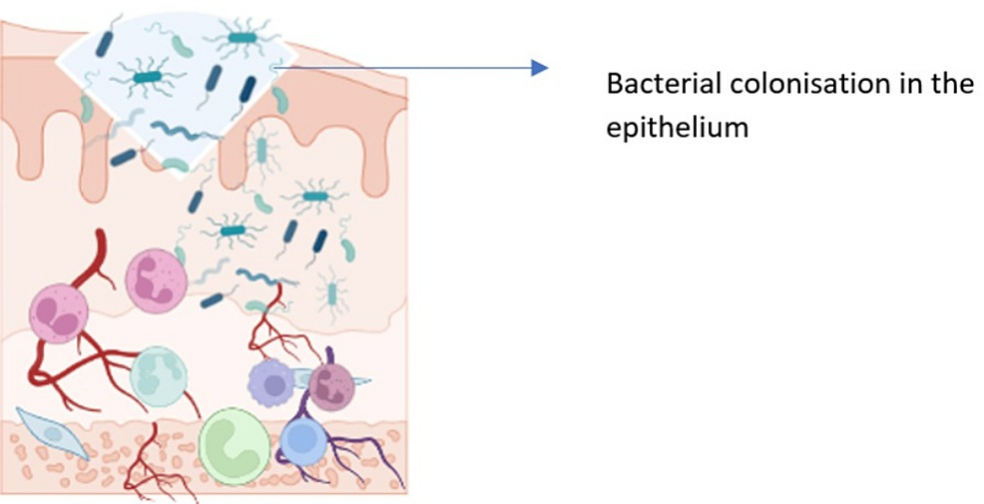
Phase IV-invasion of bacterial colonies leading to the upregulation of inflammation and tissue injury leading to ulceration Image credits: Dr. Sangavi R

Due to the upregulation of pro-inflammatory cytokines, the oral mucosa is infiltrated with a large number of inflammatory cells, which contributes to mucosal ulceration. Additionally, the byproducts of pro-inflammatory cytokines and the colonizing oral microflora can further upregulate the inflammatory response and lead to secondary infection. The inflammatory cells that infiltrate the oral mucosa in response to pro-inflammatory cytokines include neutrophils, macrophages, and lymphocytes. These cells release a variety of inflammatory mediators, including cytokines, chemokines, and proteases. These mediators further damage the mucosa and contribute to ulceration. Secondary infection is a common complication of OM, especially in patients who are neutropenic. The disruption of the mucosal barrier and the presence of inflammatory cells create an environment that is conducive to bacterial growth. Common pathogens that can cause secondary infection in patients with OM include* Pseudomonas aeruginosa*, *Staphylococcus aureus*, and *Candida albicans*. Secondary infection can further exacerbate the inflammation and tissue damage associated with OM. It can also lead to systemic complications, such as sepsis and pneumonia (Figure 4).

Phase 5: Healing

**Figure 5:**
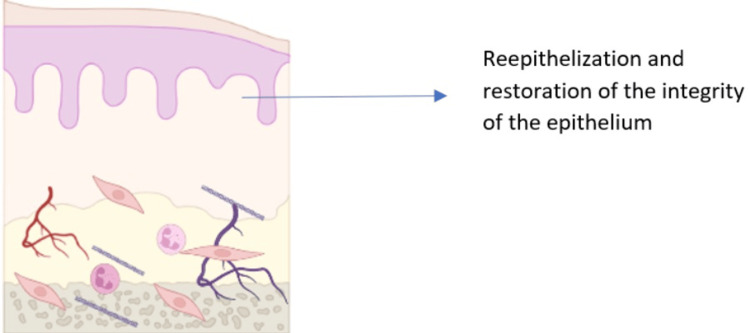
Phase V-signaling from the extracellular matrix to the epithelial cells to migrate, proliferate, and differentiate Image credits: Dr. Sangavi R

The healing phase of OM is characterized by the proliferation of epithelial cells and cellular differentiation, which restores the integrity of the epithelium. Epithelial proliferation is the process by which epithelial cells multiply. Cellular differentiation is the process by which epithelial cells mature and develop into specialized cell types, such as keratinocytes and squamous cells. The healing phase of OM is typically initiated when the pro-inflammatory response subsides. This can occur spontaneously or with treatment. Once the inflammatory response is under control, epithelial cells can begin to proliferate and differentiate, repairing the damaged mucosa. The healing process can take several weeks or even months, depending on the severity of the mucositis. It is governed by a complex network of growth factors and cytokines. The extracellular matrix plays an important role in supporting epithelial proliferation and differentiation. The oral microbiome also plays a role in healing, as some bacteria can promote epithelial regeneration. During this time, it is important to provide patients with supportive care, such as pain management and nutritional support (Figure 5).

Clinical presentation

OM typically occurs on the nonkeratinized surfaces of the oral cavity, such as the buccal and labial mucosa, lateral tongue, ventral tongue, and soft palate [4]. The onset of OM can be sudden or gradual. Patients generally complain of discomfort or pain while eating and increased bleeding while brushing. The pain associated with OM can be severe, especially when the ulcers are deep. Radiotherapy-induced OM begins after the initiation of radiation therapy to the head and neck. It starts as acute inflammation in the oral mucous membrane and lasts between seven and 98 days [7]. Chemotherapy-induced OM is typically associated with the dose of cytotoxic drug and usually develops within 1-2 weeks of the given dose [8]. It initially develops as erythema, which later progresses into erosion and ulceration. The ulcerated region is covered with a pseudomembranous layer, which eventually peels off [5]. OM in patients undergoing HSCT resolves as the absolute neutrophil count recovers. The severity of OM can vary depending on the type and dose of treatment, the patient's overall health status, and other factors. In mild cases, OM may cause only minor discomfort. In severe cases, it can lead to pain, difficulty eating and drinking, and an increased risk of infection. Patients with OM may also experience other symptoms, such as dry mouth, dysgeusia, and halitosis. In severe cases, OM can lead to nutritional deficiencies and weight loss [8].

Evaluation

The WHO scale is the most universally accepted scale for grading the severity of OM. It is a simple and easy-to-use scale that combines both subjective and objective measures.

The WHO scale is often used in clinical trials and research studies to assess the severity of OM and the efficacy of treatments. It is also used in clinical practice to guide management decisions (Table 1) [9].

**Table 1:** WHO grading of oral mucositis

Grades	Clinical presentations
Grade 0	No oral mucositis
Grade 1	Erythema and soreness
Grade 2	Ulcers, able to eat solids
Grade 3	Ulcers, requires a liquid diet (due to mucositis)
Grade 4	Ulcers, alimentation not possible (due to mucositis

In addition to the WHO scale, there are a number of other scales that can be used to grade the severity of OM. These include the Common Terminology Criteria for Adverse Events (CTCAE), the Oral Mucositis Assessment Scale (OMAS), and the Eastern Cooperative Oncology Group (ECOG) scale. However, the WHO scale is the most widely used and accepted scale.

It is important to note that the severity of OM can change over time. Therefore, it is important to reassess the severity of OM on a regular basis, especially in patients who are receiving ongoing treatment. The CTCAE Grading for OM is as follows (Table 2) [10]. 

**Table 2:** CTCAE grading for oral mucositis

Grades	Clinical presentation
Grade 1	Asymptomatic or mild symptoms; intervention not indicated
Grade 2	Moderate pain or ulcer that does not interfere with oral intake; modified diet indicated
Grade 3	Severe pain; interfering with oral intake
Grade 4	Life-threatening consequences; urgent intervention indicated
Grade 5	Death

The OMAS consists of two parameters, ulceration measured in centimeter square and the extent of erythema (Table 3) [11].

**Table 3:** The Oral Mucositis Assessment Scale

Clinical presentation	Grade			
	0	1	2	3
Ulceration/path U (cm^2^)	-	<1	1-3	>3
Erythema E	No	Moderate	Pronounced	-

Management

Oral Hygiene Protocol

Maintaining good oral hygiene is essential for preventing and managing OM. It helps to reduce the number of bacteria in the mouth, which can prevent secondary infections [12]. It also helps to remove debris and irritants from the oral cavity, which can promote healing. Good oral hygiene for patients with OM includes brushing gently with an ultrasoft toothbrush, flossing daily, and using a mouthwash, such as saline water, sodium bicarbonate rinse, or magic mouthwash. Magic mouthwash is a combination of diphenhydramine, viscous lidocaine, bismuth subsalicylate, and corticosteroid [1]. It is the most effective mouthwash for treating OM. It is important to note that patients with OM may need to modify their oral hygiene routine depending on the severity of their condition. For example, patients with severe OM may need to avoid brushing altogether and use a mouthwash only [13,14].

Pain Management

Topical anesthetics, such as lidocaine, can be effective in reducing the severity of OM lesions and relieving pain. However, the effectiveness of topical anesthetics can vary depending on the agent used. Topical anesthetics are a good option for relieving OM pain at home. Opioid analgesics, such as morphine and oxycodone, are also effective in relieving pain associated with OM. However, it is important to note that opioid analgesics can have serious side effects, such as constipation, nausea, and vomiting. Therefore, opioid analgesics should be used with caution in patients with OM. Other medications that may be used to manage OM pain include nonsteroidal anti-inflammatory drugs (NSAIDs), such as ibuprofen and acetaminophen, gabapentin, and pregabalin; and neuromodulators, such as amitriptyline and nortriptyline. The best medication for managing OM pain will depend on the individual patient's needs and response. Topical anesthetics should be used sparingly, as they can anesthetize the taste buds and make it difficult to eat and drink. Opioid analgesics should be used only as needed and at the lowest effective dose. Patients taking opioid analgesics should be monitored closely for side effects. It is important to note that there is no one-size-fits-all approach to managing OM pain. The best approach will vary depending on the individual patient's needs and response [12].

Chemoprotective Agent

Palifermin is a keratinocyte growth factor that acts as a chemoprotective agent. It is recommended for the treatment of severe OM (grade 3 or higher) in patients undergoing autologous HSCT. In these patients, palifermin has been shown to reduce the incidence and duration of severe OM. Palifermin works by stimulating the growth and differentiation of keratinocytes, which are the cells that line the oral mucosa. This helps to protect the oral mucosa from the damage caused by chemotherapy and radiation therapy. Palifermin is administered by injection under the skin once a day for seven days, starting one day before HSCT. It is generally well-tolerated, with the most common side effects being mild rash and pruritus. Palifermin is an effective treatment for severe OM in patients undergoing autologous HSCT. It can help to reduce the severity and duration of OM, which can improve the patient's quality of life and reduce the risk of complications. However, palifermin is not approved for the treatment of OM in patients who are not undergoing autologous HSCT. Palifermin is not effective in preventing OM, but it can help to reduce the severity and duration of OM in patients who develop it. The major drawback of this drug is it’s a relatively expensive medication. Overall, palifermin is an effective and safe treatment for severe OM in patients undergoing autologous HSCT. It can help to improve the patient's quality of life and reduce the risk of complications [11].

Low-Level Laser Therapy (LLLT)

LLLT is a promising new treatment for OM. It is being investigated for its ability to promote wound healing and reduce pain and inflammation. A double-blinded trial showed that LLLT was beneficial in preventing OM in patients receiving high-dose chemotherapy for HSCT [15]. LLLT is thought to work by combating the production of free radicals and pro-inflammatory cytokines, which play a role in the pathogenesis and progression of OM. LLLT is a noninvasive and painless procedure that is generally well-tolerated. It is administered using a handheld device that emits low-level laser light. The light is applied to the oral mucosa for several minutes each day for a period of several weeks. LLLT is thought to work by a number of mechanisms, including reducing inflammation, promoting angiogenesis, stimulating the proliferation and differentiation of keratinocytes, and reducing the production of free radicals. LLLT is generally safe and well-tolerated. LLLT is still under investigation, but it has the potential to be a valuable tool for the prevention and treatment of OM. Overall, It is safe and well-tolerated, and it has the potential to be effective in preventing and treating OM [13,16].

Cryotherapy

Cryotherapy is a procedure that uses cold temperatures to freeze and destroy tissues. Cryotherapy is most effective in preventing OM from developing in the first place. It is less effective in treating OM once it has already developed. Cryotherapy is typically administered before, during, and after the administration of chemotherapy. It has been shown to be effective in preventing OM in patients receiving certain chemotherapeutic agents, such as 5-fluorouracil and high-dose melphalan. Cryotherapy works by reducing blood flow to the oral mucosa, which reduces the amount of chemotherapy that reaches the tissue. This can help to protect the oral mucosa from the damage caused by chemotherapy. Cryotherapy can be administered in a variety of ways, including placing ice chips in the mouth, swishing with ice-cold water, or using a special cryotherapy device or a probe. The best way to administer cryotherapy will depend on the individual patient's needs and preferences. Cryotherapy is generally safe and well-tolerated. However, it is important to note that cryotherapy can cause side effects, such as pain, numbness, and blistering. The duration of cryotherapy treatment will vary depending on the type of chemotherapy being used and the patient's individual response. Cryotherapy is a valuable tool for preventing OM in patients receiving certain chemotherapeutic agents. It is safe and well-tolerated, and it can help to improve the patient's quality of life and reduce the risk of complications [13,17].

*Antioxidants* 

Amifostine is a radioprotective agent that acts as a scavenger of free radicals, which are thought to play a role in the initiation of OM. However, due to inadequate data, there is no consensus on the use of amifostine for the prevention of OM in patients receiving chemotherapy or radiotherapy [18]. Topical amifostine formulations containing various antioxidant agents and N-acetylcysteine have been developed. A placebo-controlled trial in patients with head and neck cancer receiving radiotherapy and chemotherapy showed that topical amifostine significantly reduced the incidence of OM at radiation doses of up to 50 Gy. Amifostine is generally well-tolerated, but it can cause side effects such as nausea, vomiting, and hypotension. However, amifostine is a relatively expensive medication. Overall, topical amifostine is a promising new treatment for the prevention of OM in patients receiving radiotherapy and chemotherapy. However, more research is needed to confirm its efficacy and safety [19].

*Anti-inflammatory Agents* 

Benzydamine hydrochloride is an NSAID that suppresses the production of inflammatory cytokines, including TNF-α. In a phase 3 trial, benzydamine hydrochloride was shown to reduce the severity of OM in patients with head and neck cancer who were undergoing radiation therapy up to a total dose of 50 Gy [20]. Based on this evidence and other literature, the Multinational Association of Supportive Care in Cancer (MASCC)/International Society of Oral Oncology (ISOO) guidelines recommend the use of benzydamine hydrochloride for the prevention of OM in patients undergoing radiation therapy. However, benzydamine hydrochloride has not been approved for this use by the US Food and Drug Administration (FDA) [10]. Additionally, many patients with head and neck cancer receive more than 50 Gy of radiation therapy, often in combination with chemotherapy. A recent phase 3 trial of benzydamine hydrochloride for the prevention of radiation-induced OM in patients with head and neck cancer was discontinued due to negative results of an interim analysis. Benzydamine hydrochloride is generally well-tolerated, but it can cause side effects such as burning and stinging in the mouth as well as nausea and vomiting. Commercially, benzydamine hydrochloride is available as a mouthwash and as a gel. The optimal dose and duration of treatment with benzydamine hydrochloride for the prevention of OM is still being investigated. Overall, benzydamine hydrochloride is a promising agent for the prevention of OM in patients undergoing radiation therapy. However, more research is needed to confirm its efficacy and safety in this setting, especially in patients receiving high-dose radiation therapy or chemotherapy [21].

Zinc Supplementation

Zinc is a mineral that plays an important role in wound healing. It helps to promote reepithelialization and scavenges free radicals. Zinc has been shown to be beneficial as adjuvant therapy in patients undergoing chemotherapy or radiation for malignancy. It can help to prevent and treat OM, a common side effect of these treatments. Zinc is most effective when started before the onset of OM. Zinc can be taken orally or administered topically. Oral zinc supplements are typically taken in doses of 25-50 mg per day. Topical zinc can be applied to the mouth as a mouthwash or gel [22]. Zinc is generally safe and well-tolerated. However, it is important to note that high doses of zinc can cause side effects such as nausea, vomiting, and diarrhea. Zinc can also be used to treat OM once it has developed. However, it is important to note that zinc is not a cure for OM, and it may not be effective in all patients. Zinc is a relatively inexpensive and easily accessible medication. Overall, zinc is a promising agent for the prevention and treatment of OM in patients undergoing chemotherapy or radiation. It is generally safe and well-tolerated, and it can help to improve the patient's quality of life [12].

Nutrition

Diet plays a crucial role in managing the flare-up of OM. Diet should be restricted to soft and bland foods that do not traumatize the oral mucosa. Foods that contain less salt and acid are also advisable. Sipping water frequently helps to combat the friction of the oral mucosa due to lack of saliva, which in turn prevents further damage. Patients are advised to avoid foods that are spicy, hot, crunchy, or hard; avoid foods that are acidic, such as citrus fruits and tomatoes; avoid foods that are salty, such as chips and pretzels; choose foods that are easy to chew and swallow; eat small, frequent meals throughout the day; and drink plenty of fluids, such as water and juice [23].

Herbal Management

While many herbal agents show promise in treating OM, their use remains a subject of discussion. These natural remedies exert various effects through mechanisms like antioxidant, pain-relieving, anti-inflammatory, antifungal, antiseptic, and even anticancer activities. Several agents, including aloe vera, honey, curcumin, Hangeshashinto, and chamomile, are used for this purpose. Among them, honey stands out with its well-documented medicinal properties, particularly in tissue repair, wound healing, and various mucosal diseases. Despite their potential, the use of herbal medicines for OM remains debatable. While some evidence suggests benefits, more research is crucial to establish their efficacy and safety definitively. Therefore, they may be better suited as complementary rather than primary treatments. Further studies are needed to solidify their role in managing this condition [24].

## Conclusions

Radiotherapy and chemotherapy are associated with a number of side effects, including OM, which can cause significant discomfort and pain for patients and impair their quality of life. While some side effects cannot be avoided, there are a number of preventive measures that can be taken to reduce their severity. The oral cavity is the most common site of discomfort and pain caused by mucositis. Oral physicians play a vital role in managing OM and providing relief to patients. In addition to providing preventive care, oral physicians can also treat OM using a variety of methods, including medications, topical therapies, nutritional support, and other therapies such as laser therapy and cryotherapy, to manage OM. Oral physicians can also work with other healthcare professionals, such as oncologists, dietitians, and nurses, to provide comprehensive care for patients with OM. They also educate patients and their caregivers about OM, including how to prevent it and manage its symptoms. It is also crucial for patients to consult other healthcare professionals for specialized care, if and when needed.
